# Circulating R-Spondin-1, Nesfatin-1, and Irisin in Gastric Cancer: A Biologically Complementary Multimarker Signature

**DOI:** 10.3390/biomedicines14102363

**Published:** 2026-10-16

**Authors:** Adem Aslan, Volkan Gökmen, Mahmut Baran Yerlikaya, Harun Bayram, Dursun Alper Yılmaz, Eva Kajti, Halit Demir

**Affiliations:** 1 Department of General Surgery, Faculty of Medicine, Ağrı İbrahim Çeçen University, Ağrı 04100, Türkiye; aslana@agri.edu.tr (A.A.); hbayram@agri.edu.tr (H.B.); 2 Department of Nursing, Faculty of Health Sciences, Ağrı İbrahim Çeçen University, Ağrı 04100, Türkiye; vgokmen@agri.edu.tr (V.G.); dursunyilmaz@uludag.edu.tr (D.A.Y.); 3 Department of General Surgery, Faculty of Medicine, Van Yüzüncü Yıl University, Van 65100, Türkiye; mahmutbaranyerlikaya@yyu.edu.tr; 4 Department of Physiology, Faculty of Medicine, Bursa Uludağ University, Bursa 16059, Türkiye; 5 Department of Nursing, Faculty of Health Sciences, Atlas University, İstanbul 34408, Türkiye; 6 Department of Chemistry, Faculty of Science, Van Yüzüncü Yıl University, Van 65080, Türkiye; halitdemir@yyu.edu.tr

**Keywords:** gastric cancer, circulating biomarkers, R-Spondin-1, nesfatin-1, irisin, multimarker signature, case–control study, exploratory discrimination

## Abstract

**Background/Objectives:** Circulating biomarkers reflecting distinct aspects of tumor and host biology may provide complementary information in gastric cancer. We characterized serum R-Spondin-1, nesfatin-1, and irisin and explored whether their combined assessment improved case–control discrimination beyond individual markers. **Methods:** In this single-center case–control study, serum concentrations were measured by ELISA in 49 patients with histopathologically confirmed gastric adenocarcinoma and 50 age- and sex-frequency-matched apparently healthy controls; all samples were obtained before any gastric resection. Between-group differences were quantified using effect sizes and 95% confidence intervals. Associations with gastric cancer status were examined using Firth penalized logistic regression; discrimination was explored using ROC analysis with bootstrap internal validation. **Results:** R-Spondin-1 was higher in gastric cancer than in controls (14.03 ± 2.75 vs. 8.47 ± 2.48 ng/mL; Cohen’s d = 2.12), whereas nesfatin-1 (2.79 ± 0.88 vs. 4.19 ± 0.81 ng/mL; d = −1.65) and irisin (7.48 ± 2.18 vs. 11.52 ± 1.97 ng/mL; d = −1.95) were lower. Associations remained after adjustment. Individual AUCs were 0.926, 0.885, and 0.913; the exploratory three-marker signature yielded an AUC of 0.964 (95% CI 0.932–0.996) and an optimism-corrected AUC of 0.958. Results were materially similar in treatment-naive patients. **Conclusions:** Gastric cancer was associated with increased R-Spondin-1 and decreased nesfatin-1 and irisin. This pattern likely reflects tumor–host interaction, including mucosal and wasting-related contributions, rather than independent tumor-specific signals. These case–control estimates do not represent diagnostic accuracy, do not support early-detection use, and require prospective validation in clinically representative cohorts.

## 1. Introduction

Gastric cancer remains a major global health burden. GLOBOCAN 2022 estimated 968,784 new stomach cancer cases and 660,175 deaths worldwide, ranking stomach cancer fifth for both incidence and cancer mortality [1]. Noninvasive biomarkers are attractive because endoscopy is invasive and resource-intensive, but the clinical value of a blood-based marker depends strongly on the target population and intended use. Conventional serum markers such as carcinoembryonic antigen (CEA) and carbohydrate antigen 19-9 (CA19-9) have limited sensitivity for early gastric neoplasia, and contemporary multimarker studies continue to use them primarily as comparators rather than adequate stand-alone detection tests [2,3]. A recent review of blood-based and liquid-biopsy strategies similarly emphasizes that promising discovery-stage accuracy often attenuates in stage I disease, precursor lesions, and clinically heterogeneous populations [4]. Accordingly, exploratory biomarker studies should distinguish biological case–control separation from validated clinical diagnostic performance.

R-Spondin-1, nesfatin-1, and irisin represent biologically distinct candidates that may provide complementary information. R-Spondin-1 potentiates Wnt/β-catenin signaling through receptors including LGR5, an axis implicated in gastric carcinogenesis and stem-cell biology. Experimental and organoid studies have linked R-Spondin/LGR5 signaling to gastric cancer proliferation, invasion, stemness, tumor growth, and Wnt dependence [5,6,7]. In the normal stomach, however, the dominant stromal niche signal is R-spondin 3 (RSPO3) produced by gastric myofibroblasts, and its expression is increased by *Helicobacter pylori* (*H. pylori*) infection [8]; circulating R-Spondin-1 therefore has to be interpreted against a background of mucosal inflammation as well as tumor biology. NUCB2/nesfatin-1 is produced predominantly by endocrine X/A-like cells of the gastric oxyntic mucosa, where NUCB2 mRNA expression is at least 10-fold higher than in the brain [9], and is involved in energy homeostasis and systemic metabolism but also participates in cancer-related cellular programs. Gastric carcinoma studies have linked NUCB2/nesfatin-1 to proliferation, migration, invasion, epithelial–mesenchymal transition, and suppression of cellular senescence [10,11,12]. Importantly, the limited human circulating literature is not directionally uniform: prior studies reported higher circulating or plasma nesfatin-1 in gastric cancer than in healthy controls [10,13], underscoring that tumor-tissue expression and circulating concentrations should not be assumed to move in parallel. Irisin, a cleavage product of FNDC5 released mainly from skeletal muscle [14], is likewise biologically heterogeneous. A recent meta-analysis found lower serum irisin across cancer populations overall [15], whereas gastric cancer studies have reported both increased and decreased circulating concentrations, together with tissue-level associations involving FNDC5/irisin [16,17,18,19,20].

Most evidence for these molecules has been generated separately, making it unclear whether they identify overlapping or complementary aspects of gastric cancer biology. Contemporary translational studies increasingly evaluate multimarker approaches in clinically relevant populations rather than relying only on healthy controls. Examples include prospective validation of a serum miRNA panel in 4566 symptomatic patients undergoing endoscopy [3], a multicenter prospective methylation cohort of 1381 participants that included gastric dysplasia and other malignancies [21], a plasma protein signature tested in separate discovery and validation cohorts [22], an independently validated multi-methylation panel [23], and real-world studies incorporating nonmetastatic disease and clinically relevant comparators [2,24]. These designs matter because apparent discrimination in highly contrasted discovery samples may not transport to the population in which a test would actually be used [4,25]. For an initial exploratory study, therefore, the most defensible question is whether a reproducible circulating pattern exists and whether the three markers provide complementary case–control information, rather than whether a clinically deployable diagnostic test has already been established.

Accordingly, this study aimed to characterize circulating R-Spondin-1, nesfatin-1, and irisin in patients with histopathologically confirmed gastric cancer and apparently healthy controls. The primary analytical objective was to quantify the magnitude of between-group differences. Secondary objectives were to examine biomarker associations with gastric cancer status after covariate adjustment and, as an exploratory analysis, to assess whether combined measurement of the three biomarkers improved case–control discrimination over individual markers. Sensitivity analyses, including restriction to treatment-naive patients, were used to examine the stability of the observed pattern.

## 2. Materials and Methods

### 2.1. Study Design and Setting

This single-center observational case–control study evaluated circulating R-Spondin-1, nesfatin-1, and irisin in patients with gastric cancer and apparently healthy controls. The study was conducted at Ağrı Training and Research Hospital, Türkiye, in academic collaboration with Ağrı İbrahim Çeçen University. Serum samples and clinical data were collected during routine clinical care between December 2025 and March 2026. The protocol, entitled “Determination of R-Spondin-1, Nesfatin-1, and Irisin Levels in Patients with Gastric Cancer”, was approved by the Ağrı İbrahim Çeçen University Scientific Research Ethics Committee on 27 November 2025 (Decision No. 505). All procedures were conducted in accordance with the Declaration of Helsinki and institutional ethical requirements. Reporting was guided by the STROBE statement for observational studies [26]. Because exploratory discrimination analyses were also performed, relevant STARD principles concerning participant selection, intended use, and limits of generalizability were used to frame their reporting [25].

### 2.2. Participants

The case group comprised adults (≥18 years) with histopathologically confirmed gastric adenocarcinoma followed in General Surgery and/or Medical Oncology. Cases were recruited consecutively among eligible patients presenting to these departments during the study period. All blood samples were obtained before any gastric resection; no patient had undergone gastrectomy, radiotherapy, targeted therapy, or immunotherapy before sampling. Patients were enrolled either before systemic therapy or during the first treatment cycle. Early treatment was defined as initiation of first-line systemic chemotherapy no more than 14 days before blood sampling, with sampling on days 8–10 of the first cycle and before the second cycle; patients with prior radiotherapy, targeted therapy, immunotherapy, or other anticancer treatment were excluded from this subgroup. Of the 20 patients who had initiated systemic therapy before sampling, 15 had metastatic disease and received first-line palliative chemotherapy, and 5 had locally advanced non-metastatic disease and had started perioperative chemotherapy with the FLOT regimen (fluorouracil, leucovorin, oxaliplatin, and docetaxel) as part of a curative-intent plan; these 5 patients were sampled during the first preoperative cycle, before surgery. The control group comprised consecutive adults presenting to the same hospital for routine health check-up who were apparently healthy, had no known malignant disease or major acute or chronic conditions expected to materially influence the biomarkers, and were frequency-matched to cases by age and sex. Controls were recruited consecutively, and enrollment into an age-sex stratum was stopped once the target proportion for that stratum had been reached. Controls did not undergo endoscopy, *H. pylori* testing, or assessment of gastric atrophy. The same exclusion framework was applied to both groups: acute infection, uncontrolled major endocrine disease, severe renal insufficiency (estimated glomerular filtration rate < 30 mL/min/1.73 m^2^), severe hepatic insufficiency (Child-Pugh class C), or other clinically significant inflammatory/systemic conditions. The sample size had been planned for the primary between-group comparison using a two-sided independent-samples *t*-test (alpha = 0.05, power = 0.80, anticipated Cohen’s d = 0.8), yielding a minimum of 40 participants per group. This calculation was not intended to establish a sample size for clinical prediction-model validation. The final analytical sample comprised 49 gastric cancer cases and 50 controls.

### 2.3. Clinical and Demographic Data Collection

Demographic and clinical variables were abstracted from hospital records and study case-report forms. Age, sex, body mass index (BMI), smoking status, hemoglobin, albumin, and C-reactive protein (CRP) were available for all participants. For patients with gastric cancer, tumor stage (I–II vs. III–IV), distant metastasis (present vs. absent), and treatment status (treatment-naive vs. early treatment) were recorded. Staging was clinical (cTNM) according to the 8th edition of the American Joint Committee on Cancer (AJCC) classification in all cases; pathological (pTNM) staging was not available because no patient had undergone resection before sampling. Tumor location (antrum, body/fundus, cardia, or diffuse involvement) was abstracted from endoscopy and pathology reports, and Lauren classification, histological grade, and the presence of a signet-ring cell component were recorded when reported in the diagnostic pathology report. HER2, mismatch-repair/microsatellite-instability (MMR/MSI), Epstein–Barr virus (EBV), and Claudin18.2 status were not systematically available. Weight-loss history, physical activity, and body composition or sarcopenia measures were not collected, and performance status was not evaluated in the present analyses. *Helicobacter pylori* status, determined by histology or urea breath testing, was available for 32 of 49 patients (65.3%). These disease-characteristic analyses were considered exploratory.

### 2.4. Biospecimen Collection and Laboratory Measurements

Peripheral venous blood samples were collected from all participants after an overnight fast (≥8 h) using standard venipuncture into serum separator tubes. Serum was separated by centrifugation at 3000× *g* for 10 min at 4 °C within 30 min of collection, aliquoted, and stored at −80 °C until analysis. Hemolysis was assessed visually and by spectrophotometric measurement of free hemoglobin (absorbance at 414 nm); a hemolysis index > 50 mg/dL was prespecified as a sample-exclusion threshold. No otherwise eligible participant exceeded this threshold, so hemolysis did not result in additional exclusions. Samples were subjected to a single freeze–thaw cycle. Serum concentrations of R-Spondin-1, nesfatin-1, and irisin were measured in duplicate using commercially available enzyme-linked immunosorbent assay (ELISA) kits according to the manufacturers’ instructions. R-Spondin-1 was measured using a kit from Cloud-Clone Corp. (Wuhan, China; Cat. No. SEA365Hu), nesfatin-1 using a kit from MyBioSource (San Diego, CA, USA; Cat. No. MBS703083), and irisin using a kit from Elabscience Biotechnology Inc. (Wuhan, China; Cat. No. E-EL-H6125). Detailed assay characteristics, including detection range, lower limit of detection, intra-assay and inter-assay coefficients of variation, sample dilution, and storage-stability information, are provided in Supplementary Table S1. Laboratory personnel were blinded to case–control status. To minimize pre-analytical variability, samples were processed using a standardized protocol, stored under identical conditions, and analyzed within the same assay batches whenever possible. These procedures reduce, but do not eliminate, pre-analytical and analytical variability. Independent inter-laboratory, cross-platform, and lot-to-lot reproducibility were not evaluated; therefore, absolute concentrations should be interpreted as assay-platform-specific measurements, and no clinical threshold is inferred from them.

### 2.5. Outcomes

The primary analytical objective was the magnitude of the between-group difference in circulating R-Spondin-1, nesfatin-1, and irisin concentrations. Secondary objectives were (i) estimation of biomarker associations with gastric cancer status after adjustment for prespecified demographic covariates and (ii) exploratory assessment of case–control discrimination for each biomarker and for a three-marker signature. Associations with tumor stage, metastasis, tumor location, histological subtype, inflammatory burden, treatment status, metabolic parameters, and *H. pylori* status were exploratory and were not interpreted as confirmatory endpoints.

### 2.6. Statistical Analysis

Statistical analyses were performed in R version 4.5.2 (R Foundation for Statistical Computing, Vienna, Austria). All statistical tests were two-sided, and a nominal *p* value < 0.05 was considered statistically significant unless a multiplicity-adjusted threshold was specified. Estimates are reported with 95% confidence intervals (CIs). Because the principal objective was to characterize the magnitude and consistency of biomarker differences rather than to establish a clinical prediction rule, interpretation prioritized effect sizes, precision of estimates, and stability across prespecified sensitivity analyses. Multiplicity adjustment was applied to the 12 planned exploratory disease-characteristic comparisons using the Bonferroni method (alpha = 0.05/12 = 0.0042); other secondary analyses were considered exploratory.

### 2.7. Data Inspection and Descriptive Analysis

Continuous variables were summarized as mean ± standard deviation (SD) when approximately normally distributed and as median with interquartile range (IQR) when skewed; categorical variables were summarized as counts and percentages. Distributional form was evaluated using histograms and Q-Q plots together with the Kolmogorov–Smirnov test. Homogeneity of variances was assessed with Levene’s test. Biomarker distributions were inspected before inferential testing and model fitting. Because circulating peptide concentrations can be right-skewed, logarithmic transformation of the three biomarkers was examined as a prespecified sensitivity analysis rather than applied automatically to the primary analysis.

### 2.8. Between-Group Comparisons

Baseline continuous variables were compared between groups using the independent-samples *t*-test when distributional assumptions were adequately met and the Mann–Whitney U test for skewed variables; categorical variables were compared using the chi-square test or Fisher’s exact test when expected cell counts were small. For the three primary biomarker comparisons, assumptions for parametric comparison were satisfied, and independent-samples *t*-tests were used. Between-group biomarker contrasts were reported as mean differences with 95% CIs and standardized effect sizes calculated as Cohen’s d using the pooled within-group SD. The magnitude and precision of these effect sizes, rather than *p* values alone, were treated as the primary quantitative description of the circulating biomarker pattern.

### 2.9. Correlation Analyses

Exploratory associations between biomarker concentrations and continuous clinical variables (age, BMI, hemoglobin, albumin, and CRP) were assessed using Pearson correlation coefficients when approximately linear parametric assumptions were met and Spearman rank correlations otherwise. Additional nesfatin-1 correlations with fasting glucose, HOMA-IR, and triglycerides were evaluated in participants with available measurements and were reported in Supplementary Table S6. Correlation analyses were interpreted descriptively and were not used for variable selection in the regression models.

### 2.10. Multivariable Logistic Regression

Binary logistic regression was used to quantify associations between biomarker concentrations and gastric cancer status. Conventional maximum-likelihood estimation showed sparse-data/separation behavior; therefore, Firth’s penalized-likelihood logistic regression was used to obtain finite odds ratios (ORs) and 95% profile-likelihood CIs [27]. Model 1 examined each biomarker separately. Model 2, the core adjusted model, included R-Spondin-1, nesfatin-1, irisin, age, and sex. Model 3, the extended sensitivity model, additionally included BMI, smoking status, hemoglobin, albumin, and CRP. Continuous predictors were entered on their original measurement scales; ORs therefore represent the change in odds per 1 ng/mL increase in each biomarker, per 1 year of age, per 1 kg/m^2^ of BMI, per 1 g/dL of hemoglobin or albumin, and per 1 mg/L of CRP. Sex was coded as male versus female and smoking as ever versus never. Covariates were prespecified on clinical and biological grounds rather than selected using univariable *p* values. Age and sex were retained because matching was by frequency rather than by individual pairs. Multicollinearity was assessed using variance inflation factors, with all VIFs < 5. Model 3 was interpreted as a robustness analysis because BMI, hemoglobin, albumin, and CRP may partly reflect disease-associated nutritional or inflammatory status. ORs were interpreted as within-sample measures of association, not as causal effects or individualized risk estimates.

### 2.11. Exploratory Discrimination Analysis

Receiver operating characteristic (ROC) analysis was used to quantify case–control discrimination for each biomarker and for a three-marker signature. Individual-marker AUCs were calculated from the observed biomarker values. The combined signature was generated from predicted probabilities of a Firth logistic regression model containing R-Spondin-1, nesfatin-1, and irisin only; demographic and clinical covariates were not included in the ROC signature. AUCs are reported with 95% CIs. Because the same participants contributed to all ROC curves, paired DeLong tests were used for exploratory comparisons of the combined signature with each individual biomarker. Internal optimism in the combined-signature AUC was assessed using 500 bootstrap resamples; the optimism-corrected AUC was obtained by subtracting the mean bootstrap-estimated optimism from the apparent AUC [28]. For descriptive threshold analysis, the Youden index (sensitivity + specificity − 1) was maximized within each bootstrap resample; the median and IQR of the resulting threshold, sensitivity, specificity, and Youden index are reported in Supplementary Table S3. These threshold estimates are sample-dependent and are not presented as validated clinical cutoffs. Positive and negative predictive values were not emphasized because the case–control sampling fraction does not estimate disease prevalence in the intended clinical population [25]. Bootstrap validation was treated solely as internal validation and not as evidence of external transportability.

### 2.12. Subgroup and Disease Characteristic Analyses

Within the gastric cancer group, exploratory two-group comparisons were performed for tumor stage (I–II vs. III–IV), metastatic status (absent vs. present), treatment status (treatment-naive vs. early treatment), and CRP strata (<10 vs. ≥10 mg/L) for each of the three biomarkers, yielding 12 planned comparisons. Biomarker concentrations are reported as mean ± SD within each subgroup. Raw *p* values are presented together with Bonferroni-adjusted *p* values; the family-wise significance threshold was alpha = 0.0042. Because subgroup sample sizes were limited, these analyses were considered hypothesis-generating regardless of nominal significance unless they remained significant after multiplicity correction. Additional descriptive comparisons by tumor location (body/fundus vs. antrum), Lauren subtype (intestinal vs. diffuse), and *H. pylori* status were outside this 12-comparison family, were restricted to cases with available data, and are reported only descriptively in the Supplementary Materials.

### 2.13. Sensitivity Analyses

Sensitivity analyses assessed whether the principal findings were materially dependent on distributional assumptions, influential observations, inflammatory extremes, treatment exposure, or age range. The analyses were repeated after (i) logarithmic transformation of biomarker concentrations, (ii) exclusion of observations exceeding ±3 SD from the corresponding group mean, (iii) exclusion of participants with CRP > 50 mg/L, (iv) restriction to treatment-naive gastric cancer cases (n = 29), and (v) restriction to participants aged 50–75 years. For each sensitivity analysis, the direction and magnitude of the adjusted biomarker ORs and the three-marker AUC were compared with the main analysis; the full results are reported in Supplementary Table S2.

### 2.14. Missing Data

Complete data were available for all 99 participants for the three biomarkers and the variables included in Models 1–3; therefore, no imputation was performed for the primary, regression, or ROC analyses. *H. pylori* status and selected metabolic variables were incomplete and were analyzed using available cases only, with the corresponding denominators reported in the Supplementary Materials. The use of apparently healthy controls creates an intentionally contrasted case–control setting; consequently, discrimination estimates quantify separation between the sampled groups and should not be generalized as clinical diagnostic accuracy in symptomatic populations, benign gastroduodenal disease, or premalignant conditions [25].

## 3. Results

### 3.1. Participant Flow

**Figure 1:**
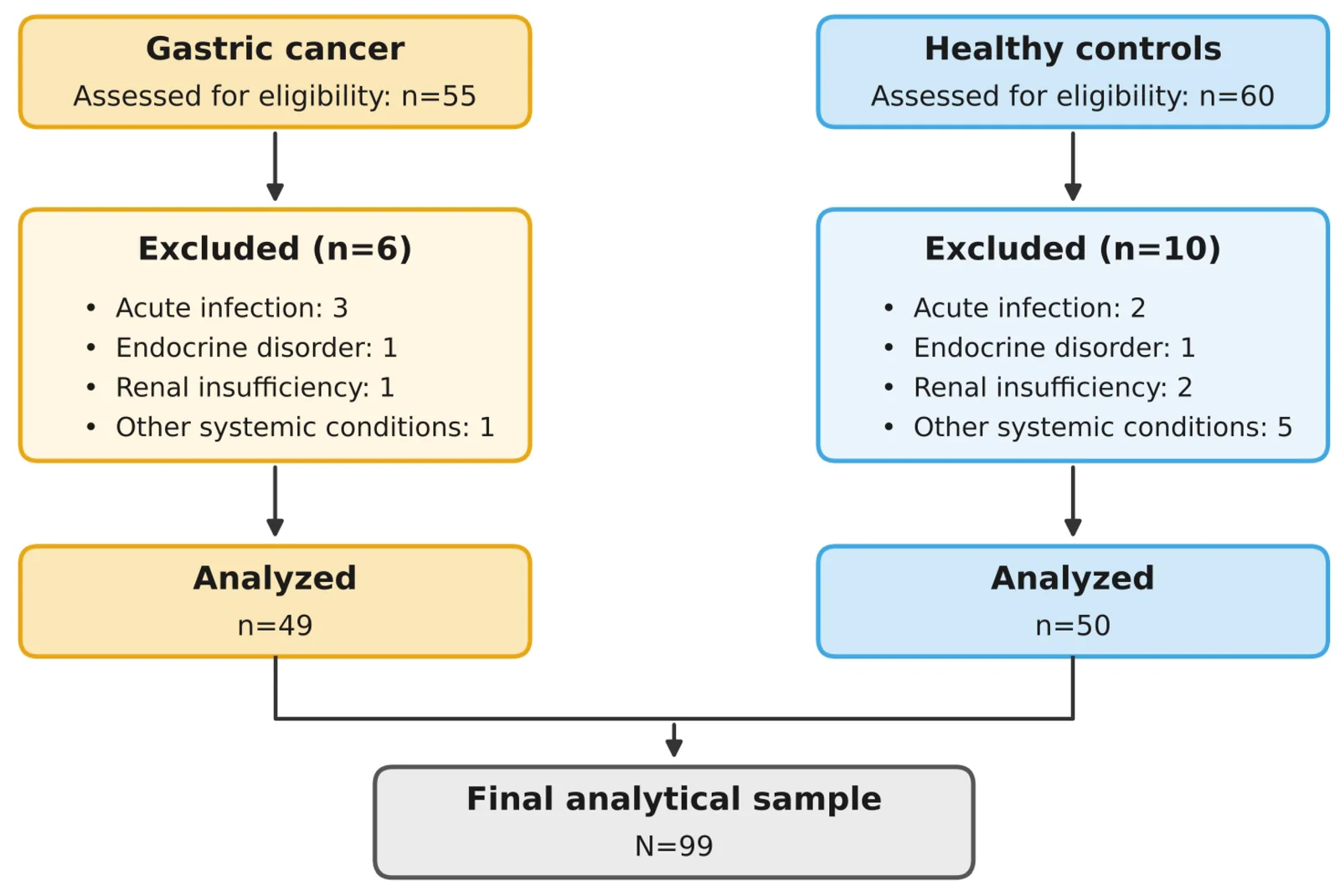
STROBE participant flow diagram. Cases and controls were recruited consecutively between December 2025 and March 2026 and screened against the same prespecified exclusion criteria.

Participant flow is shown in Figure 1 in accordance with the reporting principles of STROBE [26]. A total of 55 gastric cancer patients and 60 healthy controls were assessed for eligibility. After applying the prespecified exclusion criteria—acute infection (3 patients, 2 controls), major endocrine disorders (1, 1), severe renal insufficiency (1, 2), severe hepatic insufficiency (0, 0), and other inflammatory/systemic conditions (1, 5)—49 cancer patients and 50 controls were included in the final analytical sample. Recruitment was consecutive in both groups. Tumor stage, metastatic status, and treatment status of the analyzed cases are summarized in Table 1 and Supplementary Table S9.

### 3.2. Study Population

Baseline characteristics are presented in Table 1. Cases and controls were similar in age (62.1 ± 9.8 vs. 60.4 ± 10.2 years; *p* = 0.421) and sex distribution (59.2% vs. 60.0% male; *p* = 0.935). Patients with gastric cancer had lower BMI, hemoglobin, and albumin and higher CRP than controls (all *p* < 0.001), whereas smoking prevalence did not differ significantly. Within the gastric cancer group, 34 patients (69.4%) had stage III–IV disease, 15 (30.6%) had distant metastasis, 29 (59.2%) were treatment-naive, and 20 (40.8%) had initiated first-line systemic chemotherapy before sampling (palliative chemotherapy in 15 patients with metastatic disease and perioperative FLOT in 5 patients with non-metastatic disease). Tumor location was recorded for 45 of 49 cases (91.8%): antrum in 18 (36.7%), body/fundus in 14 (28.6%), cardia in 9 (18.4%), and diffuse involvement in 4 (8.2%) (Supplementary Table S8). All cases were sampled before any gastric resection (Supplementary Table S9). Lauren classification was available for 18 cases (36.7%; intestinal 10, diffuse 6, mixed 2) and histological grade for 12 (24.5%); a signet-ring cell component was reported in 4 cases (8.2%) (Supplementary Table S10).

### 3.3. Biomarker Distributions and Between-Group Comparisons

**Figure 2:**
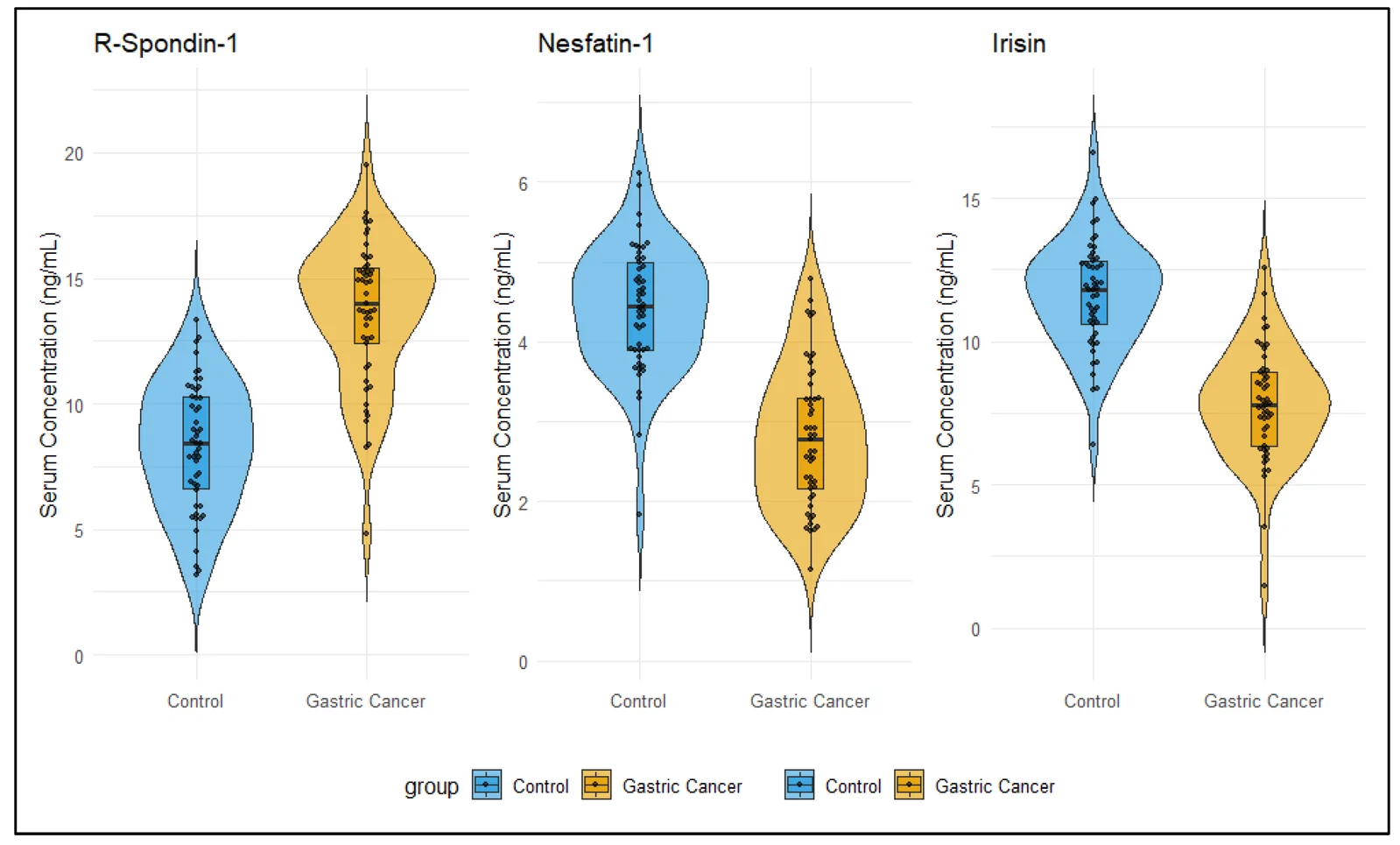
Distribution of circulating R-Spondin-1, nesfatin-1, and irisin levels in patients with gastric cancer and controls. *Violin plots with embedded box plots and overlaid individual data points display the distribution of each biomarker by study group. Central lines indicate medians, and box limits indicate the interquartile range. All between-group differences were statistically significant (p < 0.001, independent-samples t-test). Effect sizes (Cohen’s d) were 2.12 for R-Spondin-1, −1.65 for nesfatin-1, and −1.95 for irisin.*

The distributions of circulating R-Spondin-1, nesfatin-1, and irisin are summarized in Table 2 and Figure 2. Compared with controls, gastric cancer cases had higher R-Spondin-1 (14.03 ± 2.75 vs. 8.47 ± 2.48 ng/mL; mean difference 5.56 ng/mL, 95% CI 4.52–6.60; Cohen’s d = 2.12; *p* < 0.001) and lower nesfatin-1 (2.79 ± 0.88 vs. 4.19 ± 0.81 ng/mL; mean difference −1.40 ng/mL, 95% CI −1.74 to −1.06; d = −1.65; *p* < 0.001) and irisin (7.48 ± 2.18 vs. 11.52 ± 1.97 ng/mL; mean difference −4.04 ng/mL, 95% CI −4.87 to −3.21; d = −1.95; *p* < 0.001). Thus, all three biomarkers showed large between-group standardized differences, with opposite directions for R-Spondin-1 versus nesfatin-1 and irisin.

### 3.4. Independent Associations with Gastric Cancer Status

**Figure 3:**
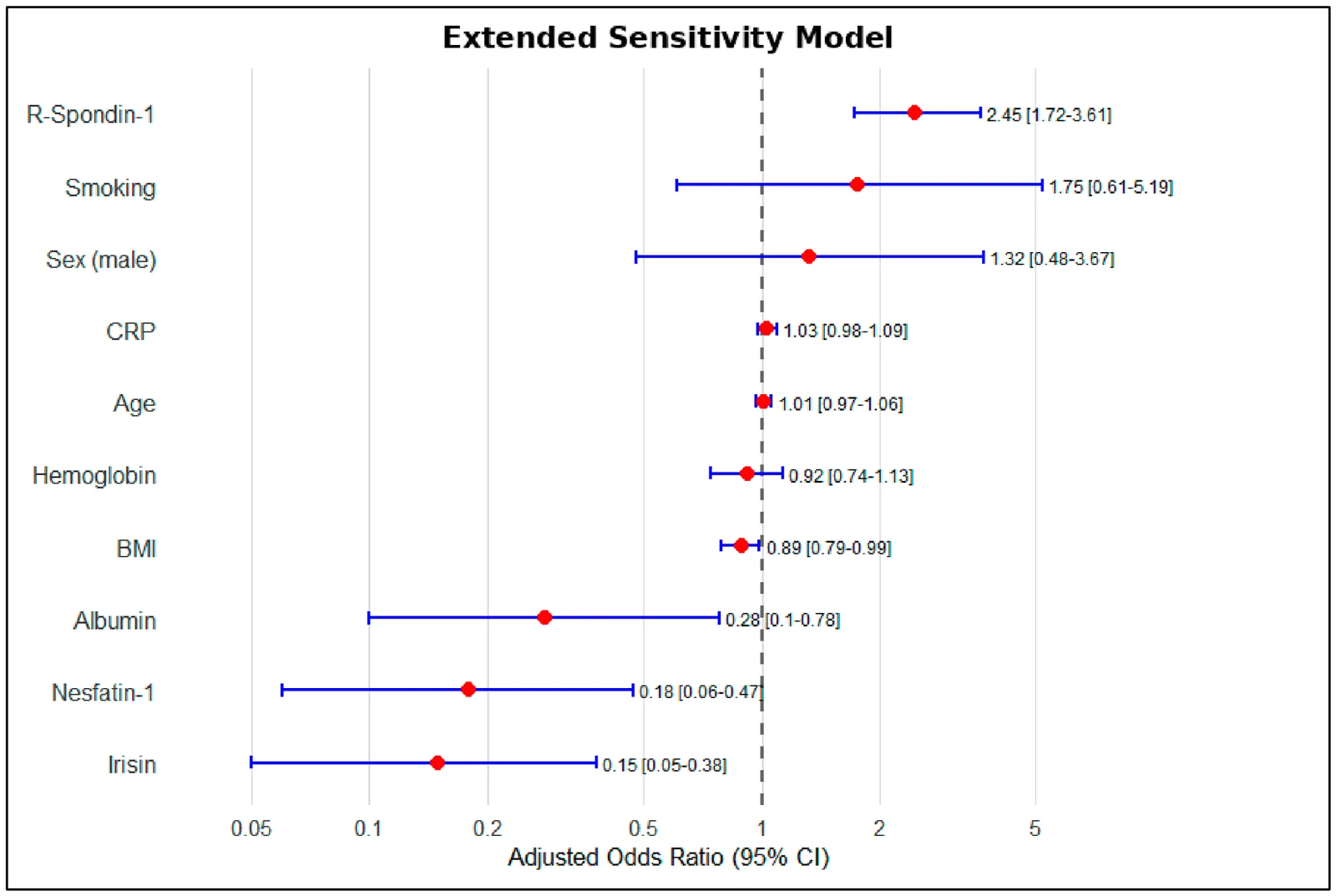
Associations between circulating biomarkers, clinical covariates, and gastric cancer status in the extended sensitivity model.

Firth penalized-likelihood regression was used because conventional maximum-likelihood models showed sparse-data/separation behavior (Table 3). In Model 1, each biomarker was strongly associated with gastric cancer status. In the core adjusted model (Model 2: all three biomarkers plus age and sex), the associations were materially unchanged. In the extended model (Model 3; Figure 3), which additionally included BMI, smoking, hemoglobin, albumin, and CRP, R-Spondin-1 remained positively associated with gastric cancer (OR = 2.45, 95% CI 1.72–3.61), while nesfatin-1 (OR = 0.18, 95% CI 0.06–0.47) and irisin (OR = 0.15, 95% CI 0.05–0.38) remained inversely associated. The stability of these estimates across increasingly adjusted models is more informative than the significance of any single covariate in this exploratory dataset. Model 3 was used to examine robustness of the biomarker associations rather than to define a deployable multivariable prediction model.

Forest plot showing odds ratios (ORs) and 95% confidence intervals (CIs) from the extended Firth penalized-likelihood model. The model includes age, sex, BMI, smoking status, hemoglobin, albumin, CRP, R-Spondin-1, nesfatin-1, and irisin. The vertical reference line denotes OR = 1. Because this extended model contains variables that may reflect disease-associated nutritional or inflammatory status, it is interpreted as a sensitivity model rather than as a clinical prediction model.

### 3.5. Exploratory Case–Control Discrimination

**Figure 4:**
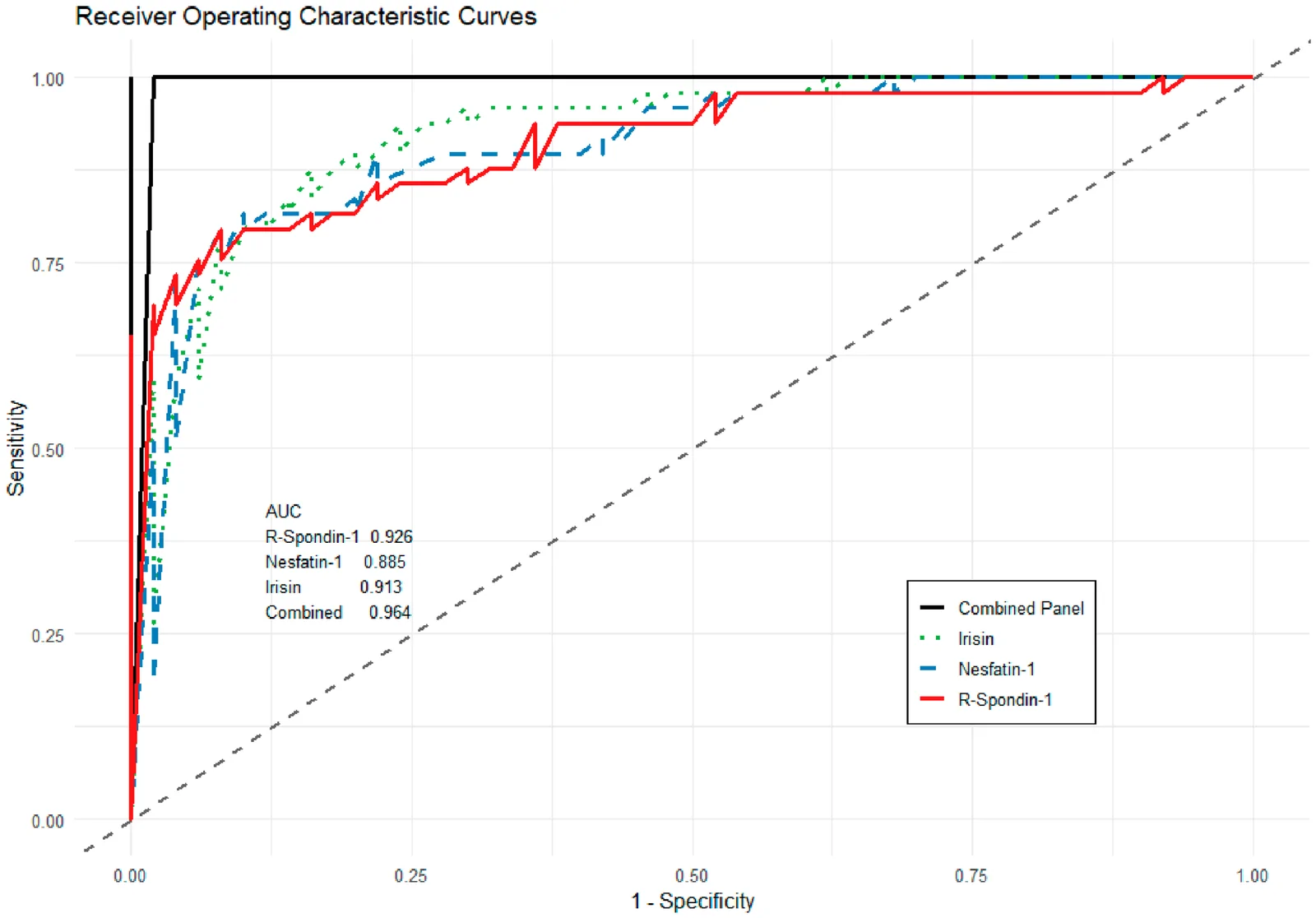
Receiver operating characteristic curves for individual biomarkers and the exploratory three-marker signature. Curves show case–control discrimination in the study sample. The combined signature yielded the highest AUC (0.964), followed by R-Spondin-1 (0.926), irisin (0.913), and nesfatin-1 (0.885). The dashed diagonal line indicates no discrimination. These AUCs should be interpreted in the context of an exploratory case–control design using apparently healthy controls.

ROC curves for the individual biomarkers and the three-marker signature are shown in Figure 4, with AUC estimates summarized in Table 4. R-Spondin-1 yielded an AUC of 0.926 (95% CI 0.873–0.978), nesfatin-1 0.885 (0.819–0.951), and irisin 0.913 (0.858–0.969). The combined three-marker signature yielded an AUC of 0.964 (95% CI 0.932–0.996). Exploratory DeLong comparisons indicated a higher AUC for the combined signature than for R-Spondin-1 (*p* = 0.009), nesfatin-1 (*p* = 0.002), or irisin (*p* = 0.011). These estimates describe discrimination between the sampled gastric cancer cases and apparently healthy controls and are not estimates of performance in an undifferentiated clinical population.

Bootstrap internal validation with 500 resamples produced an optimism-corrected AUC of 0.958 for the combined signature, compared with the apparent AUC of 0.964. The small difference indicates limited optimism within this dataset but does not substitute for independent external validation. Exploratory threshold stability is reported in Supplementary Table S3 and should not be interpreted as a clinically actionable cutoff.

### 3.6. Exploratory Disease-Characteristic Analyses

Within the gastric cancer group, unadjusted subgroup comparisons suggested higher R-Spondin-1 in stage III–IV versus stage I–II disease (raw *p* = 0.028) and in metastatic versus non-metastatic disease (raw *p* = 0.015), and lower irisin at CRP ≥ 10 mg/L (raw *p* = 0.045). However, none of the 12 subgroup comparisons remained statistically significant after Bonferroni correction (alpha = 0.0042). Accordingly, these patterns are presented only as hypothesis-generating observations in Supplementary Figure S1 and Tables S4 and S5 and are not interpreted as evidence of stage, metastasis, or inflammatory-burden associations. In additional descriptive analyses, nesfatin-1 was numerically lower in body/fundus tumors than in antral tumors (2.51 ± 0.79 vs. 3.02 ± 0.91 ng/mL; *p* = 0.105), whereas R-Spondin-1 and irisin varied little across tumor locations (Supplementary Table S8). Biomarker concentrations did not differ between intestinal- and diffuse-type tumors among the 16 cases with known subtype (all *p* > 0.20; Supplementary Table S10) or by *H. pylori* status among the 32 tested cases (Supplementary Table S7). When the case–control comparison was restricted to the 18 cases with antral tumors, nesfatin-1 remained lower than in controls (3.02 ± 0.91 vs. 4.19 ± 0.81 ng/mL; mean difference −1.17 ng/mL, 95% CI −1.63 to −0.71; d = −1.40; *p* < 0.001; Supplementary Table S8).

### 3.7. Sensitivity Analyses

Sensitivity analyses supported the stability of the principal case–control pattern (Supplementary Table S2). Log transformation, exclusion of extreme values, exclusion of participants with CRP > 50 mg/L, restriction to ages 50–75 years, and restriction to treatment-naive gastric cancer cases produced materially similar association estimates. Notably, the three-marker AUC remained 0.961 in the treatment-naive restriction (n = 29 cases), compared with 0.964 in the main analysis. This reduces concern that the overall pattern was driven solely by early treatment exposure, although residual treatment-related effects cannot be excluded.

## 4. Discussion

### 4.1. Principal Findings

This exploratory case–control study identified a coherent circulating pattern in gastric cancer, with higher R-Spondin-1 and lower nesfatin-1 and irisin than in apparently healthy controls. The standardized between-group differences were large for all three analytes (Cohen’s d = 2.12, −1.65, and −1.95, respectively), and the direction of association was preserved across the core and extended regression models and the prespecified sensitivity analyses. The three-marker signature also showed greater within-sample discrimination than any individual marker (AUC 0.964 versus 0.885–0.926), while the treatment-naive restriction produced a very similar AUC (0.961). Taken together, these findings support the presence of a consistent multimarker pattern in this cohort rather than a result driven by a single analyte or by early treatment exposure.

The interpretation must nevertheless remain proportional to the study design. The present data demonstrate biological and case–control discriminatory associations, not validated screening or diagnostic performance. This distinction is especially important because 69.4% of cases had stage III–IV disease and the comparator group consisted of apparently healthy adults. Both features increase biological contrast and may yield stronger separation than would be observed among patients undergoing evaluation for nonspecific upper-gastrointestinal symptoms, premalignant lesions, or benign gastroduodenal disease. In addition, cases and controls differed not only in tumor status but potentially also in gastric mucosal integrity, *H. pylori* exposure, and nutritional status, which were not characterized in controls; these host-level differences are relevant to all three analytes and are considered below.

### 4.2. Biological Interpretation of the Three-Marker Pattern

R-Spondin-1 showed the largest standardized difference in the present study (14.03 ± 2.75 versus 8.47 ± 2.48 ng/mL; d = 2.12). This direction is biologically compatible with experimental work implicating the R-Spondin/LGR5/Wnt axis in gastric tumor biology. Wang et al. demonstrated increased R-Spondin-1 and LGR5 expression in gastric cancer tissue and showed that R-Spondin-1 depletion reduced proliferation, migration, invasion, sphere formation, stem-cell marker expression, and tumor growth, with rescue by LGR5 overexpression [5]. These findings provide a mechanistic framework for the higher circulating R-Spondin-1 observed here. At the same time, the organoid work of Nanki et al. showed that human gastric cancers can acquire genetically and epigenetically distinct routes toward Wnt and R-spondin niche independence [6]. Thus, a higher serum R-Spondin-1 concentration should not be interpreted as evidence that all tumors remain functionally R-Spondin-dependent. Rather, it may reflect perturbation of a pathway that is heterogeneous across gastric cancer subtypes. Because the present study did not pair serum measurements with tumor-tissue expression, the cellular source of circulating R-Spondin-1 cannot be established.

An important alternative explanation concerns the gastric mucosal environment rather than the tumor itself. In the stomach, the principal stromal niche factor is RSPO3 rather than R-Spondin-1: Sigal et al. showed that RSPO3 produced by gastric myofibroblasts beneath the antral glands maintains Axin2-positive and Lgr5-positive epithelial stem cells, and that *H. pylori* infection increases stromal RSPO3 expression, expands the Axin2-positive cell pool, and causes gland hyperproliferation and hyperplasia [8]. *H. pylori* extracts have also been shown to induce RSPO3 and to promote cancer stem cell-like properties in gastric cancer cells through GNG7/β-catenin signaling [29], and stromal RSPO3 has been linked to T-cell infiltration, interferon-γ signaling, and PD-L1 expression in *H. pylori*-driven precancerous lesions, with tumor RSPO3 expression associated with mucosal T-cell infiltration in human intestinal-type gastric cancer [30]. The present assay measured R-Spondin-1, not RSPO3, and R-spondin family members differ in tissue distribution and receptor usage; nevertheless, these data show that *H. pylori*-associated mucosal inflammation can perturb R-spondin signaling independently of malignancy. This is relevant in Türkiye, where a nationally representative ^13^C-urea breath-test survey found *H. pylori* infection in 82.5% of adults, with the highest regional prevalence (88.1%) in the east of the country [31]. In the present cohort, 21 of the 32 tested cases (65.6%) were *H. pylori*-positive, and R-Spondin-1 did not differ significantly by *H. pylori* status among tested cases (14.11 ± 2.72 vs. 13.94 ± 2.68 ng/mL; *p* = 0.864; Supplementary Table S7); however, the status was unknown for 17 cases (34.7%) and for all controls, and no control underwent endoscopy or atrophy assessment. Part of the case–control difference in R-Spondin-1 may therefore reflect *H. pylori*-associated mucosal inflammation rather than tumor biology, and the present data cannot apportion the two contributions.

Wnt-pathway alterations are also not uniform across gastric cancer subtypes. Gastric adenocarcinomas segregate into Epstein–Barr virus-positive, microsatellite-unstable, genomically stable, and chromosomally unstable molecular subtypes with distinct pathway alterations [32], and truncating RNF43 mutations, which remove a negative-feedback brake on Wnt receptors and directly involve the R-spondin–LGR–RNF43 axis, cluster in MSI-high tumors: Jo et al. detected RNF43 frameshift mutations in 66.7% of MSI-high gastric cancers, compared with 1 of 90 microsatellite-stable/MSI-low gastric and colorectal cancers [33]. Because HER2, MMR/MSI, EBV, and Claudin18.2 statuses were not systematically available, and Lauren classification and grade were reported for only 18 and 12 cases, respectively (Supplementary Table S10), the present study cannot determine whether circulating R-Spondin-1 elevation is concentrated in a particular histological or molecular subtype. The absence of a difference between intestinal- and diffuse-type tumors in the small subgroup with known subtype should not be taken as evidence of subtype independence.

The nesfatin-1 finding is more discordant with the previous gastric-cancer literature and therefore deserves separate consideration. We observed lower serum nesfatin-1 in cases than controls (2.79 ± 0.88 versus 4.19 ± 0.81 ng/mL; d = −1.65). By contrast, Wang et al. reported higher plasma nesfatin-1 in 40 patients with gastric cancer than in 40 healthy controls (1.19 versus 0.89 ng/mL), with an AUC of 0.857, 70% sensitivity, and 95% specificity [13]. Ren et al. likewise found increased NUCB2/nesfatin-1 in gastric cancer tissue and serum and reported an AUC of 0.970, while functional knockdown reduced proliferation, migration, invasion, and epithelial–mesenchymal transition [10]. More recently, Ishibashi et al. showed that high tumor NUCB2 expression was associated with deeper invasion, lymphovascular invasion, nodal metastasis, advanced stage, and unfavorable progression-free survival, and that NUCB2 loss induced a senescence phenotype in gastric cancer cells [11]. The contrast between these tissue and prior circulating observations, and the present lower serum concentration, argues against treating circulating nesfatin-1 as a simple surrogate of intratumoral NUCB2 activity.

Several non-mutually exclusive explanations could underlie this discrepancy. The prior studies used different populations, sample matrices, ELISA platforms, and clinical spectra, and one used plasma rather than serum [10,13]. A biologically important explanation is the anatomical source of circulating nesfatin-1. NUCB2/nesfatin-1 is produced predominantly by endocrine X/A-like cells of the oxyntic mucosa, where it co-localizes with ghrelin and where NUCB2 mRNA expression is at least 10-fold higher than in the brain [9]. Circulating nesfatin-1 may therefore fall when functional fundic/corpus mucosa is replaced by tumor, damaged by atrophic gastritis or *H. pylori*, or removed at gastrectomy. Gastrectomy can be excluded as an explanation in the present cohort because all samples were obtained before resection (Supplementary Table S9). Consistent with a contribution of oxyntic mucosal loss, nesfatin-1 was numerically lowest in body/fundus tumors, where oxyntic mucosa is concentrated, and higher in antral tumors (2.51 ± 0.79 vs. 3.02 ± 0.91 ng/mL; *p* = 0.105; Supplementary Table S8), although this difference was not statistically significant in these small subgroups. Conversely, nesfatin-1 remained markedly lower than in controls even among patients with antral tumors (3.02 ± 0.91 vs. 4.19 ± 0.81 ng/mL; d = −1.40; *p* < 0.001), in whom fundic/corpus mucosa is not directly replaced by tumor. This argues against tumor replacement of oxyntic mucosa as the sole explanation, although it is not conclusive, because *H. pylori*-associated atrophic gastritis frequently extends beyond the antrum in patients with antral cancer and could still reduce functional oxyntic mass. Because serum pepsinogen I/II ratios, gastrin-17, and histological atrophy grading were not obtained in either group, the contribution of oxyntic atrophy cannot be quantified. In addition, patients with gastric cancer had lower BMI, hemoglobin, and albumin and higher CRP than controls. The inverse nesfatin-1 association persisted in the extended sensitivity model, but because these covariates may partly lie on the pathway from tumor to host deterioration, this adjustment does not exclude confounding by mucosal or nutritional status, and residual confounding and assay-specific effects remain possible. Low serum nesfatin-1 in this cohort is therefore best interpreted as a possible marker of reduced functional oxyntic mucosa and altered host metabolic state rather than as a direct readout of tumor NUCB2 activity. Replication with atrophy markers, harmonized pre-analytical procedures, and direct serum-tissue pairing will be required before the direction of circulating nesfatin-1 in gastric cancer can be considered established.

Irisin provides a second example of substantial between-study heterogeneity. Our study found lower serum irisin in gastric cancer (7.48 ± 2.18 versus 11.52 ± 1.97 ng/mL; d = −1.95) with an individual AUC of 0.913. This direction agrees with Temur and Rashid, who reported lower irisin in 21 gastric-cancer patients than in 20 controls and an AUC of 0.94 [18], and with a recent cancer-wide meta-analysis in which circulating irisin was lower overall in patients with cancer (pooled SMD −1.16) [15]. Conversely, Shahidi et al. observed higher serum irisin in 22 newly diagnosed gastric-cancer patients than in 29 controls [17]. Tissue-level data are also complex: Xu et al. reported lower FNDC5 expression in gastric cancer tissue and showed that FNDC5 overexpression inhibited migration and invasion, yet lower FNDC5 expression was not uniformly associated with worse survival across datasets [20]. These inconsistencies reinforce the need to distinguish circulating concentration, tissue expression, and functional signaling, and they also caution against transporting absolute ELISA cutoffs across laboratories or populations.

A further consideration is that irisin is a myokine: it is cleaved from FNDC5 and released mainly by skeletal muscle in a PGC-1α-dependent manner [14]. Lower circulating irisin in cancer may therefore reflect reduced muscle mass or activity as much as tumor biology. Cancer cachexia is defined by ongoing loss of skeletal muscle mass that cannot be fully reversed by conventional nutritional support [34], and the case group showed several features compatible with a wasting phenotype, including lower BMI, albumin, and hemoglobin and higher CRP (Supplementary Table S11). BMI is a poor surrogate for muscle mass; weight-loss history, physical activity, and body composition or sarcopenia measures were not collected; and performance status was not evaluated in the present analyses. Moreover, BMI and albumin may lie on the causal pathway from tumor to wasting to reduced irisin, so their inclusion in Model 3 does not remove this confounding and may partly adjust away the pathway of interest. The low irisin observed here is therefore plausibly, at least in part, a host wasting signal rather than a tumor-intrinsic marker.

### 4.3. Complementarity and Translational Context

The rationale for combining the three analytes is therefore not that they represent repeated measurements of the same process, nor that they constitute three independent tumor-derived signals. In light of the considerations above, the three markers are better viewed as capturing different components of tumor–host interaction: R-Spondin-1 as a Wnt/LGR5-related signal that may originate from the tumor, the inflamed mucosa, or both; nesfatin-1 as a marker partly dependent on functional oxyntic mucosa; and irisin as a marker more likely to reflect host muscle metabolism and cancer-associated wasting. In the present dataset, the three biomarkers moved in different directions and retained associations when modeled together, which is consistent with partially nonredundant information. The increase in AUC from 0.885 to 0.926 for the individual markers to 0.964 for the three-marker signature supports complementary within-sample discrimination, but this gain should not be read as additive tumor-specific information: part of the separation may derive from host-level differences in mucosal integrity, *H. pylori* exposure, and nutritional status that would also be present in many patients with benign gastric disease or nongastric wasting. Moreover, the magnitude of this gain should not be interpreted as proof of clinical incremental value because the signature was developed and internally assessed in the same small case–control sample [28,35].

The contrast with earlier single-marker studies is informative. Wang et al. reported an AUC of 0.857 for plasma nesfatin-1 [13], while Temur and Rashid reported an AUC of 0.94 for serum irisin in a small gastric-cancer/healthy-control comparison [18]. These values show that strong apparent discrimination can occur even for single analytes when cases are contrasted with healthy participants. Our higher combined AUC may therefore reflect both genuine complementarity and the favorable spectrum of the current sample. This is particularly relevant because most of our cases were advanced stage. Moreover, R-Spondin-1 was nominally higher in stage III–IV than in stage I–II disease (14.48 ± 2.71 vs. 13.12 ± 2.54 ng/mL; raw *p* = 0.028) and in metastatic than in non-metastatic disease (14.92 ± 2.78 vs. 13.65 ± 2.62 ng/mL; raw *p* = 0.015) (Supplementary Tables S4 and S5). Although these differences did not survive Bonferroni correction, their direction fits a tumor-burden effect, in which circulating R-Spondin-1 rises with disease extent rather than marking early tumor development, and this pattern argues against the use of R-Spondin-1, or of the present signature, for early detection. The present study was not powered to establish stage I–II performance, and any early-detection application would require dedicated validation in patients with stage I–II disease and precursor lesions.

Larger gastric-cancer biomarker studies illustrate what happens when the clinical spectrum becomes more realistic. In the multicenter study by So et al., a 12-miRNA panel had AUCs of 0.93 and 0.92 in retrospective discovery and verification cohorts, but performance decreased to 0.848 in a prospective cohort of 4566 symptomatic patients undergoing endoscopy; notably, the panel still outperformed CEA and CA19-9 in that setting [3]. Feng et al. observed a similar attenuation for a 13-protein plasma signature: early-gastric-cancer discrimination decreased from an AUC of 0.998 in discovery to 0.818 in validation [22]. Shen et al. similarly used targeted serum proteomics to propose a protein signature for resectable gastric cancer, illustrating the same discovery-to-validation pathway [36]. Long et al. reported an AUC of 0.9585 for a five-marker methylation model in training and 0.8868 in an independent validation cohort, with lower discrimination in the earliest pathological strata than in later stages [23]. These examples are directly relevant to the present AUC of 0.964: they show that internal or discovery-phase performance can remain scientifically valuable while still overestimating what will be achieved after independent, clinically representative validation.

Comparator selection is equally important. Nie et al. evaluated methylated SEPTIN9 and RNF180 across a multicenter population that included gastric dysplasia and non-neoplastic gastric conditions rather than only healthy controls [21]. Lu et al. prospectively combined a gastric-cancer screening score with serum biomarkers in 106 gastric-cancer and 143 non-cancer participants that included superficial gastritis, atrophic gastritis, and intestinal metaplasia; the validation AUC was 0.967 [37]. Du et al. went further by testing CFL1 and TAGLN2 not only against healthy controls but also against 143 patients with other non-gastric diseases or malignancies and then in a prospective cohort of 88 gastroenterology patients with unknown cancer status before sampling; they also benchmarked the new markers against conventional serum markers [24]. These designs answer a clinically harder question than the present case–control comparison: whether a biomarker distinguishes gastric cancer from the differential diagnoses encountered before endoscopic confirmation.

Accordingly, the next validation step for the present signature should not simply reproduce another gastric-cancer-versus-healthy-control cohort. It should preferentially enroll treatment-naive incident cases together with symptomatic and disease controls, including *H. pylori*-associated gastritis, peptic ulcer disease, atrophic gastritis, intestinal metaplasia, dysplasia, and other gastrointestinal malignancies. CEA and CA19-9 should be measured in parallel so that incremental value can be quantified against existing practice. The three-marker formula and intended clinical role should be prespecified before validation, and performance should be reported separately for early-stage disease. Such studies should also record *H. pylori* status and markers of gastric atrophy (e.g., pepsinogen I/II ratio and gastrin-17) in all participants, tumor location and histological and molecular subtype in cases, and weight loss, performance status, and an objective body composition or sarcopenia measure, so that the tumor and host components of the signature can be separated; measuring RSPO3 alongside R-Spondin-1 would further clarify the source of the R-spondin signal. An independent laboratory or at least a second assay lot/platform would also help determine whether the observed concentrations and ranking are analytically transportable. These features would move the work from biological discovery and internal discrimination toward clinically interpretable validation [2,3,21,23,24,25,35,37].

### 4.4. Limitations

Several considerations should be taken into account when interpreting these findings. The study was conducted at a single center with a relatively modest sample, and the sample-size calculation was designed primarily to detect between-group biomarker differences rather than to develop or validate a clinical prediction model. The sample of 49 cases and 50 controls exceeded the prespecified requirement for this primary comparison, and Firth penalized estimation and bootstrap internal validation were used to limit small-sample bias and optimism; nevertheless, confidence intervals for subgroup estimates were wide, and the sample was insufficient for reliable stage-, location-, or subtype-specific inference. In addition, the use of apparently healthy controls provided a clear biological contrast but does not fully reflect the more heterogeneous populations encountered in routine practice, where benign gastroduodenal disorders, premalignant lesions, and other causes of gastrointestinal symptoms would also need to be distinguished from gastric cancer. For this reason, the observed discrimination should be viewed as an estimate of case–control separation rather than as a direct measure of real-world diagnostic performance [25].

Treatment exposure represents another relevant consideration. No patient had undergone gastrectomy before sampling, so surgical resection did not confound the measurements; however, 20 of 49 patients had started first-line systemic chemotherapy within 14 days before sampling, including 15 patients with metastatic disease receiving palliative chemotherapy and 5 patients with non-metastatic disease who had started perioperative FLOT chemotherapy. Although none of these patients had undergone surgery, treatment-related effects on appetite, nutrition, and muscle metabolism cannot be excluded. All 20 were excluded from the treatment-naive restriction, which produced materially unchanged findings (29 cases; three-marker AUC 0.961 vs. 0.964); this mitigation does not eliminate the concern. Several host-level factors were incompletely characterized. Controls did not undergo endoscopy, *H. pylori* testing, or atrophy assessment; *H. pylori* status was missing for 17 cases (34.7%) and for all controls; and markers of oxyntic atrophy were not measured in either group. Histopathological data were incomplete (Lauren classification in 18 cases and grade in 12), and HER2, MMR/MSI, EBV, and Claudin18.2 status were not available. Weight-loss history, physical activity, and sarcopenia measures were not recorded, and performance status was not evaluated in relation to biomarker levels; cancer-associated wasting therefore cannot be fully separated from tumor-specific effects on irisin. Conventional markers such as CEA and CA19-9 were not available for direct comparison. Likewise, the exploratory subgroup analyses were limited by sample size and did not remain significant after correction for multiple testing, so they should be regarded as hypothesis-generating rather than confirmatory. Finally, because the biomarkers were measured using commercial ELISA platforms, absolute concentrations may vary across assay systems, and independent cross-platform and inter-laboratory validation will be important. Taken together, these considerations define the next step for translation: prospective external validation in clinically representative cohorts with full characterization of mucosal, infectious, and nutritional status.

## 5. Conclusions

Gastric cancer was associated with a distinct circulating pattern characterized by increased R-Spondin-1 and decreased nesfatin-1 and irisin, and the overall pattern was stable across sensitivity analyses, including restriction to treatment-naive patients. The three biomarkers provided complementary case–control information, but this complementarity most plausibly reflects tumor–host interaction, including possible contributions from *H. pylori*-associated mucosal signaling, loss of functional oxyntic mucosa, and cancer-associated wasting, rather than three independent tumor-specific signals. The nominal increase in R-Spondin-1 with advanced stage and metastasis is consistent with a tumor-burden effect and does not support early-detection use. These findings support further investigation of the multimarker signature but do not establish clinical diagnostic accuracy. Independent validation in prospective cohorts that include benign and premalignant gastric conditions, *H. pylori* and atrophy assessment, body composition measures, conventional biomarker comparators, and stage-specific analyses is required before clinical utility can be assessed.

## Figures and Tables

**Table 1:** Baseline demographic and clinical characteristics of patients with gastric cancer and control subjects.

Variable	Gastric Cancer (n = 49)	Controls (n = 50)	*p* Value
**Age, years**	62.1 ± 9.8	60.4 ± 10.2	0.421
**Sex, male, n (%)**	29 (59.2)	30 (60.0)	0.935
**Body mass index, kg/m^2^**	23.0 ± 3.2	26.1 ± 3.5	<0.001
**Smoking, n (%)**	20 (40.8)	15 (30.0)	0.291
**Hemoglobin, g/dL**	12.0 ± 1.5	14.2 ± 1.3	<0.001
**Albumin, g/dL**	3.50 ± 0.49	4.21 ± 0.31	<0.001
**C-reactive protein, mg/L**	8.1 (3.9–15.2)	2.0 (1.1–3.4)	<0.001
**Disease characteristics**			
**Tumor stage III–IV, n (%)**	34 (69.4)	—	—
**Metastasis present, n (%)**	15 (30.6)	—	—
**Treatment-naïve, n (%)**	29 (59.2)	—	—

*Data are mean ± SD, median (IQR) or n (%). p values from independent-samples t-test, Mann–Whitney U-test, χ^2^ test, or Fisher’s exact test, as appropriate.*

**Table 2:** Distribution of circulating R-Spondin-1, nesfatin-1, and irisin in patients with gastric cancer and controls.

Biomarker	Gastric Cancer (n = 49)	Controls (n = 50)	Difference	95% CI	Cohen’s d	*p* Value
**R-Spondin-1**	14.03 ± 2.75	8.47 ± 2.48	5.56	[4.52, 6.60]	2.12	<0.001
**Nesfatin-1**	2.79 ± 0.88	4.19 ± 0.81	−1.40	[−1.74, −1.06]	−1.65	<0.001
**Irisin**	7.48 ± 2.18	11.52 ± 1.97	−4.04	[−4.87, −3.21]	−1.95	<0.001

All differences are mean differences. Effect size: Cohen’s d. Independent-samples *t*-tests were used after assessment of distributional form and variance homogeneity.

**Table 3:** Logistic regression models examining the association of circulating biomarkers with gastric cancer status (Firth’s penalized likelihood).

Variable	Model 1 Single Biomarker OR (95% CI)	Model 2 Core Adjusted OR (95% CI)	Model 3 Extended Sensitivity OR (95% CI)
**R-Spondin-1 (per ng/mL)**	2.52 (1.78–3.68)	2.48 (1.74–3.65)	2.45 (1.72–3.61)
**Nesfatin-1 (per ng/mL)**	0.16 (0.05–0.45)	0.17 (0.06–0.46)	0.18 (0.06–0.47)
**Irisin (per ng/mL)**	0.14 (0.04–0.37)	0.14 (0.05–0.38)	0.15 (0.05–0.38)
**Age (per year)**	—	1.01 (0.97–1.06)	1.01 (0.97–1.06)
**Sex (male vs. female)**	—	1.35 (0.49–3.72)	1.32 (0.48–3.67)
**BMI (per kg/m^2^)**	—	—	0.89 (0.79–0.99)
**Smoking (ever vs. never)**	—	—	1.75 (0.61–5.19)
**Hemoglobin (per g/dL)**	—	—	0.92 (0.74–1.13)
**Albumin (per g/dL)**	—	—	0.28 (0.10–0.78)
**CRP (per mg/L)**	—	—	1.03 (0.98–1.09)

Model 1: each biomarker separately. Model 2: core adjusted model including all three biomarkers, age, and sex. Model 3: extended sensitivity model, additionally including BMI, smoking, hemoglobin, albumin, and CRP. All models used Firth penalized likelihood; continuous-variable ORs are per 1-unit increase.

**Table 4:** Exploratory case–control discrimination of individual biomarkers and the three-marker signature.

Biomarker/Signature	AUC	95% CI	Optimism-Corrected AUC
R-Spondin-1	0.926	0.873–0.978	-
Nesfatin-1	0.885	0.819–0.951	-
Irisin	0.913	0.858–0.969	-
Three-marker signature	0.964	0.932–0.996	0.958

AUC: area under the receiver operating characteristic curve; CI: confidence interval. The optimism-corrected AUC was estimated by 500 bootstrap resamples for the combined signature only. No clinical cutoff is proposed.

## Data Availability

The de-identified participant-level data and analysis code underlying the findings are not publicly deposited because of ethical and participant-confidentiality considerations. They are available from the corresponding author upon reasonable request, subject to applicable institutional and ethics requirements.

## Supplementary Materials

The following supporting information can be downloaded at https://www.mdpi.com/article/10.3390/biomedicines14102363/s1: Supplementary Figure S1 and Supplementary Tables S1–S11, including ELISA characteristics, sensitivity analyses, exploratory threshold stability, disease-characteristic subgroup data, multiplicity-adjusted subgroup results, metabolic correlations, *H. pylori*-stratified biomarker concentrations, biomarker concentrations by tumor location, treatment and surgical status at sampling, histopathological characteristics, and cachexia-relevant clinical variables.

## Abbreviations

AJCC, American Joint Committee on Cancer; AUC, area under the curve; BMI, body mass index; CA19-9, carbohydrate antigen 19-9; CEA, carcinoembryonic antigen; CI, confidence interval; CRP, C-reactive protein; cTNM, clinical tumor-node-metastasis classification; EBV, Epstein–Barr virus; ELISA, enzyme-linked immunosorbent assay; EMT, epithelial–mesenchymal transition; FLOT, fluorouracil, leucovorin, oxaliplatin, and docetaxel; FNDC5, fibronectin type III domain-containing protein 5; *H. pylori*, *Helicobacter pylori*; HER2, human epidermal growth factor receptor 2; IQR, interquartile range; LGR5, leucine-rich repeat-containing G-protein-coupled receptor 5; MMR, mismatch repair; MSI, microsatellite instability; NUCB2, nucleobindin-2; OR, odds ratio; ROC, receiver operating characteristic; RSPO3, R-spondin 3; SD, standard deviation; STARD, Standards for Reporting Diagnostic Accuracy Studies; STROBE, Strengthening the Reporting of Observational Studies in Epidemiology.
