# Supplementary material for: Circulating R-Spondin-1, Nesfatin-1, and Irisin in Gastric Cancer: A Biologically Complementary Multimarker Signature
Source: Biomedicines. 2026 Oct 16;14(10):2363. doi: 10.3390/biomedicines14102363 (PMC13702425; doi:10.3390/biomedicines14102363)
Supplement: Supplementary file 1 [file biomedicines-14-02363-s001.zip › biomedicines-4608484-supplementary.pdf]

Supplementary Table S1. ELISA assay characteristics (extended)

Biomarker	Catalog No.	Lot Number	Detection range	Sensitivity (LLOD)	Intra-assay CV	Inter-assay CV	Sample dilution	Storage stability at -80 °C (months)	Hemolysis exclusion threshold (mg/dL)
**R-Spondin-1**	SEA365Hu	20240115	0.1–50 ng/mL	0.05 ng/mL	3.5%	6.2%	1:2	>24	>50
**Nesfatin-1**	MBS703083	20240210	0.05–20 ng/mL	0.02 ng/mL	2.8%	5.1%	1:2	>24	>50
**Irisin**	E-EL-H6125	20240305	0.5–100 ng/mL	0.25 ng/mL	4.1%	7.0%	1:2	>24	>50

LLOD: lower limit of detection; CV: coefficient of variation. Storage-stability information is based on the manufacturers' specifications. All study samples underwent one freeze-thaw cycle; internal pilot testing assessed three freeze-thaw cycles in six samples. The hemolysis threshold was defined spectrophotometrically at 414 nm. Kits: Cloud-Clone Corp. (R-Spondin-1), MyBioSource (nesfatin-1), and Elabscience (irisin).

Supplementary Table S2. Sensitivity analyses of adjusted biomarker associations and exploratory three-marker discrimination

Analysis	R-Spondin-1 OR (95% CI)	Nesfatin-1 OR (95% CI)	Irisin OR (95% CI)	Three-marker signature AUC
**Main analysis**	2.45 (1.72–3.61)	0.18 (0.06–0.47)	0.15 (0.05–0.38)	0.964
**Log-transformed biomarkers**	2.38 (1.68–3.52)	0.20 (0.07–0.49)	0.16 (0.06–0.39)	0.961
**Outliers excluded (±3 SD)**	2.41 (1.70–3.55)	0.19 (0.06–0.48)	0.15 (0.05–0.37)	0.962
**CRP >50 mg/L excluded**	2.43 (1.69–3.58)	0.18 (0.06–0.46)	0.14 (0.04–0.36)	0.963
**Treatment-naïve only (n=29 cases)**	2.41 (1.69–3.55)	0.19 (0.06–0.49)	0.14 (0.04–0.39)	0.961
**Age restricted (50–75 years)**	2.50 (1.71–3.66)	0.17 (0.05–0.46)	0.14 (0.04–0.37)	0.966

ORs are from the extended Firth logistic regression model adjusted for age, sex, BMI, smoking, hemoglobin, albumin, and CRP. The reported AUC describes case-control discrimination in the corresponding sensitivity dataset.

Supplementary Table S3. Bootstrap stability of the sample-dependent exploratory Youden threshold (500 resamples)

Metric	Value (Median, IQR)
Exploratory Youden threshold (predicted probability)	0.48 (0.45–0.52)
Sensitivity at exploratory threshold	0.91 (0.86–0.95)
Specificity at exploratory threshold	0.91 (0.87–0.94)
**Youden index**	0.82 (0.78–0.85)

Bootstrap resampling with replacement (n=500). The threshold maximized the Youden index within each resample and is presented only to describe threshold stability in this study sample. It is not an externally validated or clinically actionable cutoff.

Supplementary Figure S1. Exploratory biomarker patterns by tumor stage, metastatic status, and CRP category.

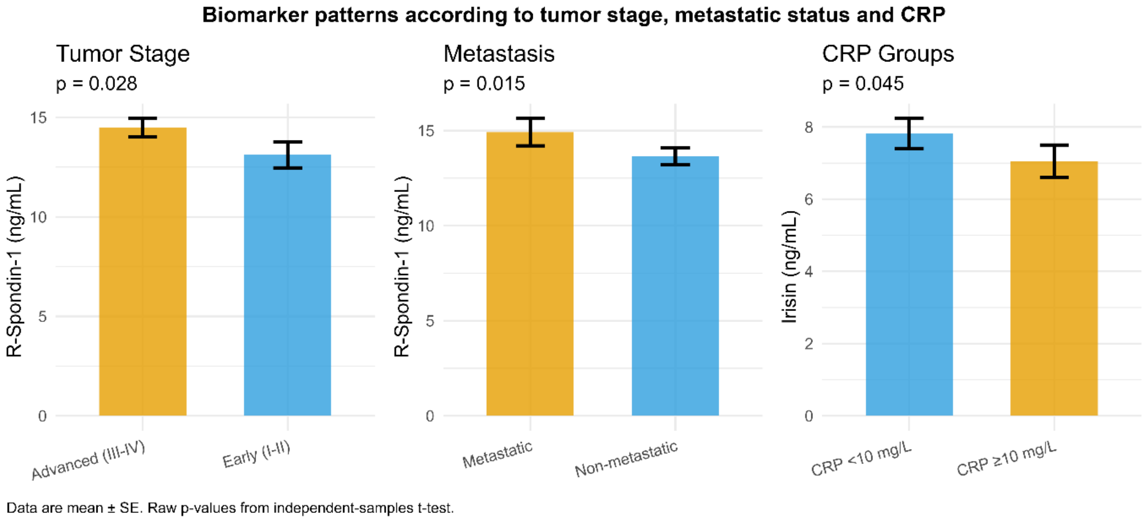

Raw p values are displayed for descriptive purposes. After Bonferroni correction for 12 subgroup comparisons, none remained statistically significant. The figure is hypothesis-generating and should not be interpreted as confirmatory evidence of stage, metastatic, or inflammatory-burden associations.

Supplementary Table S4. Biomarker concentrations according to disease-related characteristics in the gastric cancer group.

Clinical characteristic	n	R-Spondin-1 (mean ± SD)	p	Nesfatin-1 (mean ± SD)	p	Irisin (mean ± SD)	p
**Tumor stage**							
**Early (I–II)**	15	13.12 ± 2.54	0.028	2.88 ± 0.91	0.368	7.98 ± 2.24	0.087
**Advanced (III–IV)**	34	14.48 ± 2.71		2.74 ± 0.87		7.24 ± 2.10	
**Metastasis**							
**Non-metastatic**	34	13.65 ± 2.62	0.015	2.82 ± 0.89	0.521	7.67 ± 2.18	0.124
**Metastatic**	15	14.92 ± 2.78		2.70 ± 0.86		7.02 ± 2.11	
**CRP**							
**<10 mg/L**	28	13.81 ± 2.68	0.293	2.83 ± 0.90	0.587	7.82 ± 2.20	0.045
**≥10 mg/L**	21	14.32 ± 2.81		2.73 ± 0.85		7.05 ± 2.07	
**Treatment status**							
**Treatment-naïve**	29	13.95 ± 2.73	0.684	2.81 ± 0.89	0.792	7.51 ± 2.19	0.567
**Early-treatment**	20	14.15 ± 2.78		2.76 ± 0.87		7.43 ± 2.17	

Values are mean ± SD. Raw p values are shown for transparency; multiplicity-adjusted results are provided in Supplementary Table S5.

Supplementary Table S5. Bonferroni-corrected p-values for exploratory subgroup analyses (k=12 comparisons)

Comparison	Biomarker	Raw p	Bonferroni-corrected p (×12)	Significant after correction?
**Advanced stage (III–IV) vs. early (I–II)**	R-Spondin-1	0.028	0.336	No
**Advanced stage vs. early**	Nesfatin-1	0.368	1.000	No
**Advanced stage vs. early**	Irisin	0.087	1.000	No

Comparison	Biomarker	Raw p	Bonferroni-corrected p (×12)	Significant after correction?
**Metastasis present vs. absent**	R-Spondin-1	0.015	0.180	No
**Metastasis present vs. absent**	Nesfatin-1	0.521	1.000	No
**Metastasis present vs. absent**	Irisin	0.124	1.000	No
**CRP ≥10 vs. <10 mg/L**	R-Spondin-1	0.293	1.000	No
**CRP ≥10 vs. <10 mg/L**	Nesfatin-1	0.587	1.000	No
**CRP ≥10 vs. <10 mg/L**	Irisin	0.045	0.540	No
**Treatment-naïve vs. early-treatment**	R-Spondin-1	0.684	1.000	No
**Treatment-naïve vs. early-treatment**	Nesfatin-1	0.792	1.000	No
**Treatment-naïve vs. early-treatment**	Irisin	0.567	1.000	No

Bonferroni correction threshold: α=0.05/12=0.0042. None of the 12 subgroup comparisons remained statistically significant after correction; all subgroup findings are hypothesis-generating.

Supplementary Table S6. Correlation of nesfatin-1 with metabolic parameters

Parameter	n	Correlation coefficient (Spearman's ρ)	p-value
**Fasting glucose (mg/dL)**	41	–0.11	0.492
**HOMA-IR**	38	–0.08	0.621
**Triglycerides (mg/dL)**	44	0.05	0.752

Metabolic variables were available only for subsets of participants. These correlations are exploratory; no statistically significant associations were observed.

Supplementary Table S7. Biomarker levels by H. pylori status (gastric cancer group only)

H. pylori status	n	R-Spondin-1 (mean ± SD)	Nesfatin-1 (mean ± SD)	Irisin (mean ± SD)
**Positive**	21	14.11 ± 2.72	2.81 ± 0.89	7.45 ± 2.15
**Negative**	11	13.94 ± 2.68	2.76 ± 0.86	7.52 ± 2.20
**p-value**	—	0.864	0.879	0.934

H. pylori status was available for 32 of 49 patients (65.43%). No statistically significant between-group differences were observed; this incomplete-case subgroup analysis is exploratory.

Supplementary Table S8. Tumor location and circulating biomarker concentrations in the gastric cancer group (n=49).

Tumor location	n (%)	R-Spondin-1 (ng/mL)	Nesfatin-1 (ng/mL)	Irisin (ng/mL)
**Antrum**	18 (36.7)	13.87 ± 2.69	3.02 ± 0.91	7.52 ± 2.21
**Body/fundus**	14 (28.6)	14.31 ± 2.84	2.51 ± 0.79	7.39 ± 2.14
**Cardia**	9 (18.4)	14.02 ± 2.71	2.68 ± 0.82	7.61 ± 2.23
**Diffuse/linitis plastica**	4 (8.2)	14.88 ± 2.92	2.44 ± 0.76	7.18 ± 2.09
**Not recorded**	4 (8.2)	—	—	—

Values are mean ± SD. Tumor location was abstracted from endoscopy and pathology reports. Body/fundus vs. antrum, nesfatin-1: 2.51 vs. 3.02 ng/mL, $t(30)=1.67$, $p=0.105$ (independent-samples t-test). Antral tumors vs. controls (n=50; 4.19 ± 0.81 ng/mL), nesfatin-1: mean difference -1.17 ng/mL (95% CI -1.63 to -0.71), Cohen's d=-1.40, $t(66)=-5.09$, $p<0.001$; because H. pylori-associated atrophy may extend beyond the antrum, this comparison does not exclude a contribution of oxyntic mucosal loss. These descriptive comparisons were not part of the 12-comparison Bonferroni family and are hypothesis-generating. Markers of oxyntic atrophy (serum pepsinogen I/II ratio, gastrin-17) and histological atrophy grading were not available.

Supplementary Table S9. Treatment and surgical status at blood sampling in the gastric cancer group (n=49).

Status at sampling	n (%)	Definition
**No surgery, no systemic therapy**	29 (59.2)	Incident, treatment-naive
**No surgery, metastatic disease (M1), first-line palliative chemotherapy started ≤14 days before sampling**	15 (30.6)	Sampling on days 8–10 of cycle 1 and before cycle 2
**No surgery, locally advanced non-metastatic disease (M0), perioperative chemotherapy (FLOT) started ≤14 days before sampling**	5 (10.2)	Curative-intent; sampled during the first preoperative cycle, before surgery
**Prior gastrectomy**	0 (0.0)	—

Status at sampling	n (%)	Definition
**Prior radiotherapy, immunotherapy, or targeted therapy**	0 (0.0)	—

All blood samples were obtained before any gastric resection. All 20 patients who had started systemic therapy were excluded from the treatment-naive sensitivity analysis (Supplementary Table S2). FLOT: fluorouracil, leucovorin, oxaliplatin, and docetaxel. Staging was clinical (cTNM, AJCC 8th edition) in all cases; no pathological staging was available at sampling.

Supplementary Table S10. Histopathological characteristics of the gastric cancer group (n=49).

Characteristic	n (%)	Data availability
**Lauren classification**		18/49 (36.7%)
**Intestinal**	10 (20.4)	
**Diffuse**	6 (12.2)	
**Mixed**	2 (4.1)	
**Not reported (adenocarcinoma NOS)**	31 (63.3)	
**Histological grade**		12/49 (24.5%)
**Well/moderately differentiated**	5 (10.2)	
**Poorly differentiated**	7 (14.3)	
**Not reported**	37 (75.5)	
**Signet-ring cell component reported**	4 (8.2)	—
**TNM edition**	AJCC 8th	All cases
**Staging modality**	Clinical (cTNM)	All cases; no pTNM
**HER2, MMR/MSI, EBV, Claudin18.2**	Not available	—

Exploratory comparison of intestinal vs. diffuse subtype (n=16 with known subtype) showed no significant differences in R-Spondin-1, nesfatin-1, or irisin (all p>0.20); this subgroup is too small for inference. NOS: not otherwise specified; AJCC: American Joint Committee on Cancer; MMR/MSI: mismatch repair/microsatellite instability; EBV: Epstein–Barr virus.

Supplementary Table S11. Cachexia-relevant clinical variables in patients with gastric cancer and controls.

Variable	Gastric cancer (n=49)	Controls (n=50)	p value
**BMI, kg/m²**	23.0 ± 3.2	26.1 ± 3.5	<0.001
**Albumin, g/dL**	3.50 ± 0.49	4.21 ± 0.31	<0.001
**Hemoglobin, g/dL**	12.0 ± 1.5	14.2 ± 1.3	<0.001
**CRP, mg/L**	8.1 (3.9–15.2)	2.0 (1.1–3.4)	<0.001
**Weight-loss history**	Not recorded	Not recorded	—
**Performance status**	Not evaluated in this analysis	Not assessed	—
**Physical activity**	Not recorded	Not recorded	—
**Body composition/sarcopenia measure**	Not recorded	Not recorded	—

Data are mean ± SD or median (IQR). BMI, albumin, and CRP may lie on the causal pathway between tumor burden, wasting, and circulating irisin; adjustment for these variables therefore does not remove confounding by cancer-associated wasting.
